# WISP2 promotes cell proliferation via targeting ERK and YAP in ovarian cancer cells

**DOI:** 10.1186/s13048-020-00687-8

**Published:** 2020-07-25

**Authors:** Zi-Qing Shi, Zi-Yan Chen, Yao Han, Heng-Yan Zhu, Meng-Dan Lyu, Han Zhang, Yi Zhang, Liu-Qing Yang, Wei-Wei Pan

**Affiliations:** School of Medicine, Jiaxing University, Jiaxing, 314001 China

**Keywords:** Ovarian cancer, Proliferation, Apoptosis, WISP2

## Abstract

**Background:**

Wnt-inducible signaling pathway protein 2 (WISP2) is a wnt1-induced signaling pathway protein 2. Although studies indicate that WISP2 may promote the development of various tumors, its role in ovarian cancer remains unclear. The objective of the current study was to analyze the effects of WISP2 on the proliferation and migration of ovarian cancer cells in vitro and in vivo.

**Results:**

Immunohistochemistry and western blotting indicated that WISP2 was highly expressed in various ovarian cancer tissues and cell lines, but weakly expressed in normal ovary tissue. WISP2 deletion inhibited cell growth, clone formation, and migration of ovarian cancer cells while promoting cell apoptosis and affecting the cell cycle. This growth inhibitory effect caused by WISP2 loss is due to the inhibition of phosphorylated extracellular signal-related kinase (p-ERK)1/2, as well as CCAAT/enhancer-binding protein α (CEBPα) and CEPBβ. In addition, WISP2 deletion also activated the Yes-associated protein (YAP).

**Conclusion:**

WISP2 deletion inhibits ovarian cancer cell proliferation by affecting ERK signaling pathways.

## Background

Epithelial ovarian cancer (EOC) is the fifth leading cause of all female cancer-related deaths in the USA, with approximately 22,000 new cases and 16,000 deaths per year. It is the most prevalent and lethal of all gynecologic cancers [1]. Primary treatment includes surgery and platinum-based chemotherapy. In spite of treatments such as ovarian cytoreductive surgery combined with effective postoperative chemotherapy, prognosis remains unsatisfactory, and mortality statistics have changed only slightly [2]. Therefore, molecular target screening of ovarian cancer is felt to be particularly important.

Wnt-inducible signaling pathway protein 2(WISP2)is a member of the CCN protein family, which comprises six members, including CCN1 (CYR61), CCN2 (CTGF), CCN3 (NOV), CCN4 (WISP1), CCN5 (WISP2), and CCN6 (WISP3) [3–5]. Previous studies have revealed that CCNs exhibit different expression profiles and transcript levels in different tissues, organs, and tumors [6]. The six CCN members are associated with numerous cellular functions as well as pathological conditions [7]. WISP2 is a member of the cysteine-rich CCN family of extracellular matrix-associated proteins [8]. Emerging studies have demonstrated that, in cancers, WISP2 is involved in cell proliferation, migration, and metastasis [9]. However, the function of WISP2 in ovarian cancer cells remains largely unclear.

Our previous studies have indicated that WISP2 is induced by LATS1/2 deletion in MC38 cells and affected cell growth and apoptosis [10]. In the current study, we found that WISP2 was highly expressed in various ovarian cancer tissues and cell lines but weakly expressed in human normal ovary tissue. To determine the role of WISP2 in ovarian cancer, we deleted WISP2 using the CRISPR/Cas9 method. WISP2 deletion inhibited the growth, clone formation, and cell migration of ovarian cancer cells. Furthermore, WISP2 deletion promoted cell apoptosis and affected the cell cycle. These growth inhibitory effects of WISP2 loss were due to the inhibition of the phosphorylated extracellular signal-related kinase (p-ERK)1/2, as well as CCAAT/enhancer-binding protein *α* (CEBPα) and CEBPβ. In addition, WISP2 deletion also activated the Yes-associated protein (YAP). The current study revealed the potential of WISP2 as a factor capable of promoting ovarian cancer cell proliferation and survival.

## Results

### WISP2 is overexpressed in human ovarian cancer tissues and cell lines

**Fig. 1:**
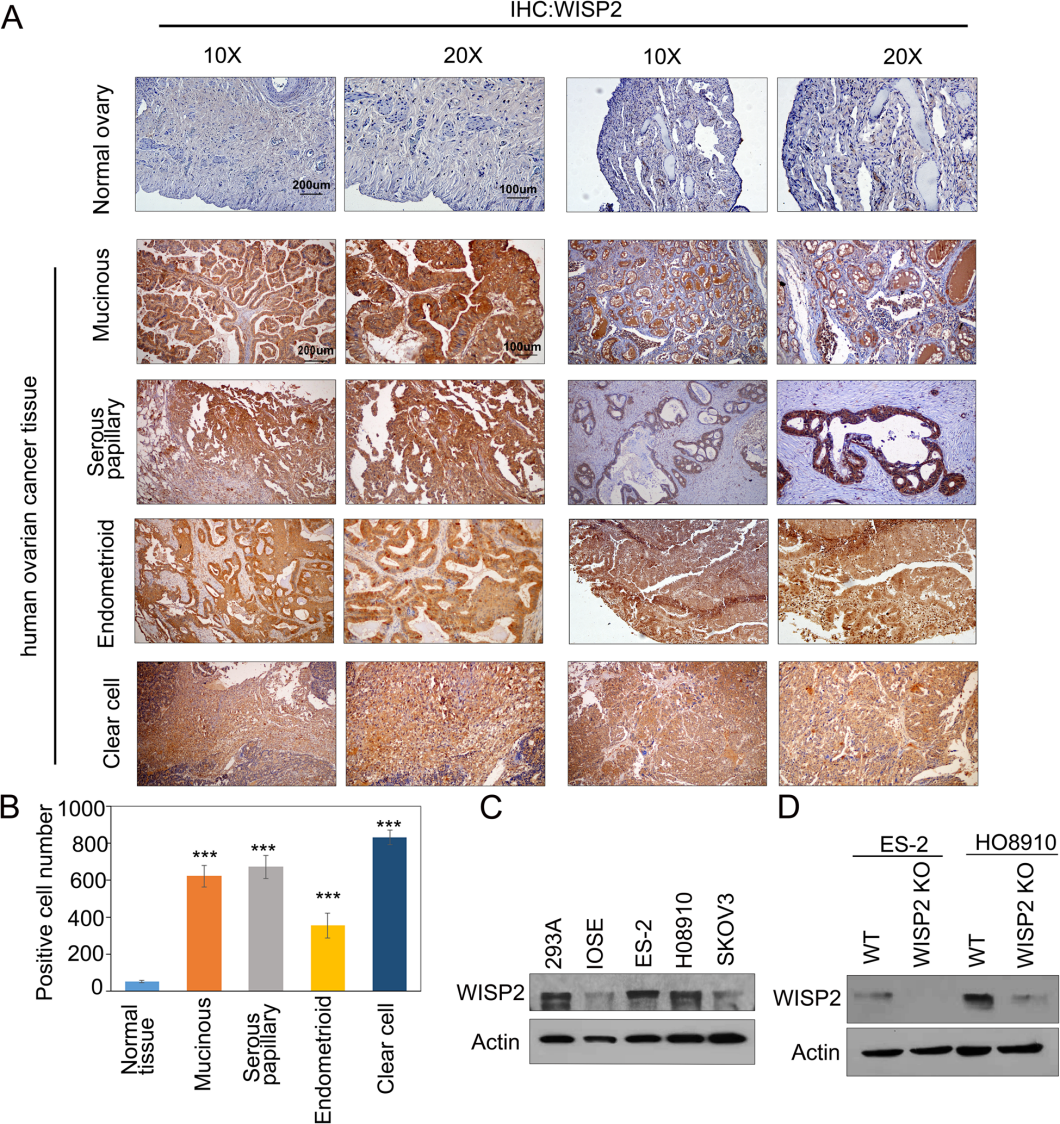
WISP2 expression patterns in human ovarian cancer samples and cell lines. **a** Immunohistochemistry results of WISP2 expression in normal ovary tissue(*n* = 3) and ovarian cancer tissues(*n* = 20). Sections were counterstained with hematoxylin and eosin; scale bars: 200 μm (10x) and 100 μm (20x). **b** Semi-quantitative statistics for IHC assays. ***p < 0.001. **c** Western blot results showing high WISP2 expression in ovarian cancer cells. **d** WISP2 knockout (KO) cells were subjected to immunoblot analysis with WISP2 and actin antibodies

WISP2 protein expression in human normal ovary tissues and ovarian cancer tissues was assessed via immunohistochemistry. WISP2 was weakly expressed in normal ovarian tissue(*n* = 3). Significant WISP2 staining intensity was detected in endometrioid carcinoma, serous cystadenoma, clear cell carcinoma, and mucinous cystadenoma tissues(*n* = 20) (Fig. 1a and b). The expression of WISP2 in ovarian cancer cell lines and immortalized ovarian surface epithelial (mOSE) cells was also examined. WISP2 was highly expressed in most ovarian cancer cell lines, such as ES-2 and HO8910 (Fig. 1c), but was weakly expressed in mOSE cells. High expression of WISP2 protein in ovarian cancer tissues and cell lines suggest that WISP2 may play an essential role in ovarian tumor cell proliferation and migration.

### WISP2 deletion inhibits cell growth and cell migration

To verify this assumption, we deleted WISP2 in the ovarian cancer cell lines ES-2 and HO8910 using CRISPR/Cas9 technology. By evaluating the protein expression of WISP2, we confirmed the successful generation of complete WISP2 knockout (KO) clones from the ES-2 cell line. However, it was somewhat difficult to obtain complete WISP2 KO clones from HO8910 cells (Fig. 1d).

**Fig. 2:**
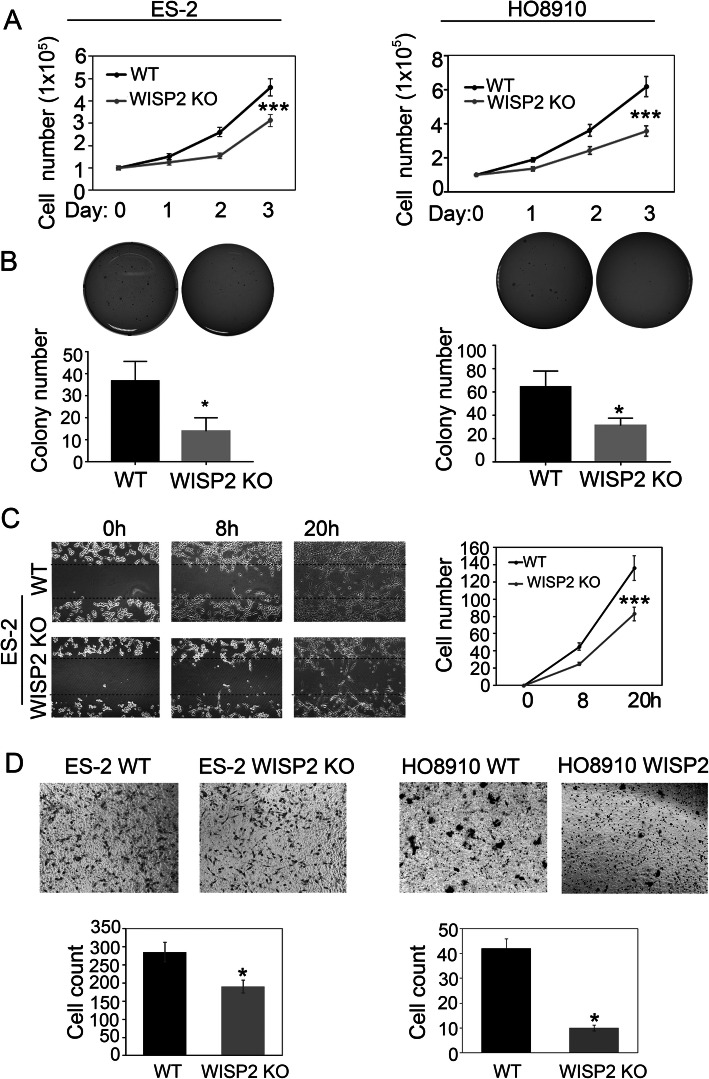
WISP2 deletion represses cell proliferation and migration. **a** WISP2 deletion inhibits ovarian cancer cell growth. ES-2 and HO8910 WT and WISP2 deletion cells (1 × 10^5^) were plated in 6-well culture dishes, and viable cells were counted following trypan blue staining. **b** WISP2 deletion inhibits anchorage-independent growth of ES-2 and HO8910 cells. Cells were cultured in Matrigel. Colonies that formed on days 12 and 17 are shown. Colony numbers were counted on days 14 to 21. **c** Wound-healing assay for the migration capability of ES-2 WT and WISP2 deletion cells. **d** Transwell test to detect the migration capability of ES-2 and HO8910 WT and WISP2 deletion cells. Cells were added to transwells and allowed to migrate for 12 h. Cells at the upper surface of the membrane were removed with cotton swabs, and cells on the bottom surface were stained with hematoxylin and eosin. The experiment was replicated thrice. Error bars represent the standard deviation (s.d.). *p < 0.05, **p < 0.01, ***p < 0.001

We tested whether knocking out WISP2 inhibits the growth of ovarian cancer cells. For this reason, we compared the cell growth between wild-type (WT) and WISP2 KO cells by cell growth assay. WISP2 deletion significantly suppressed cell growth in ES-2 and HO8910 cells (Fig. 2a). We also examined anchorage-independent growth of WT and WISP2 KO cells via a soft agar assay and found that WISP2 deletion strongly inhibited anchorage-independent growth in ES-2 and HO8910 cells (Fig. 2b). Next, we analyzed the expression of the cancer cell proliferation markers KI-67 and p-Histone H3 using immunofluorescence and western blot assay. WISP2 deletion significantly inhibited KI-67 and p-Histone H3 protein expression in ES-2 and HO8910 cells (Fig. 4a and b).

The effect of WISP2 on ovarian cancer cell migration was also determined. A scratch wound assay demonstrated that WISP2 deletion noticeably inhibited the migration of ES-2 cells (Fig. 2c). In addition, we examined the effect of WISP2 deletion on the metastatic potential of ovarian cancer ES-2 and HO8910 cells using a transwell assay. The migration of the cells was decreased upon WISP2 deletion (Fig. 2d). These results demonstrate that WISP2 is required for ovarian cancer cell proliferation and migration.

### WISP2 deletion promotes apoptosis and senescence

**Fig. 3:**
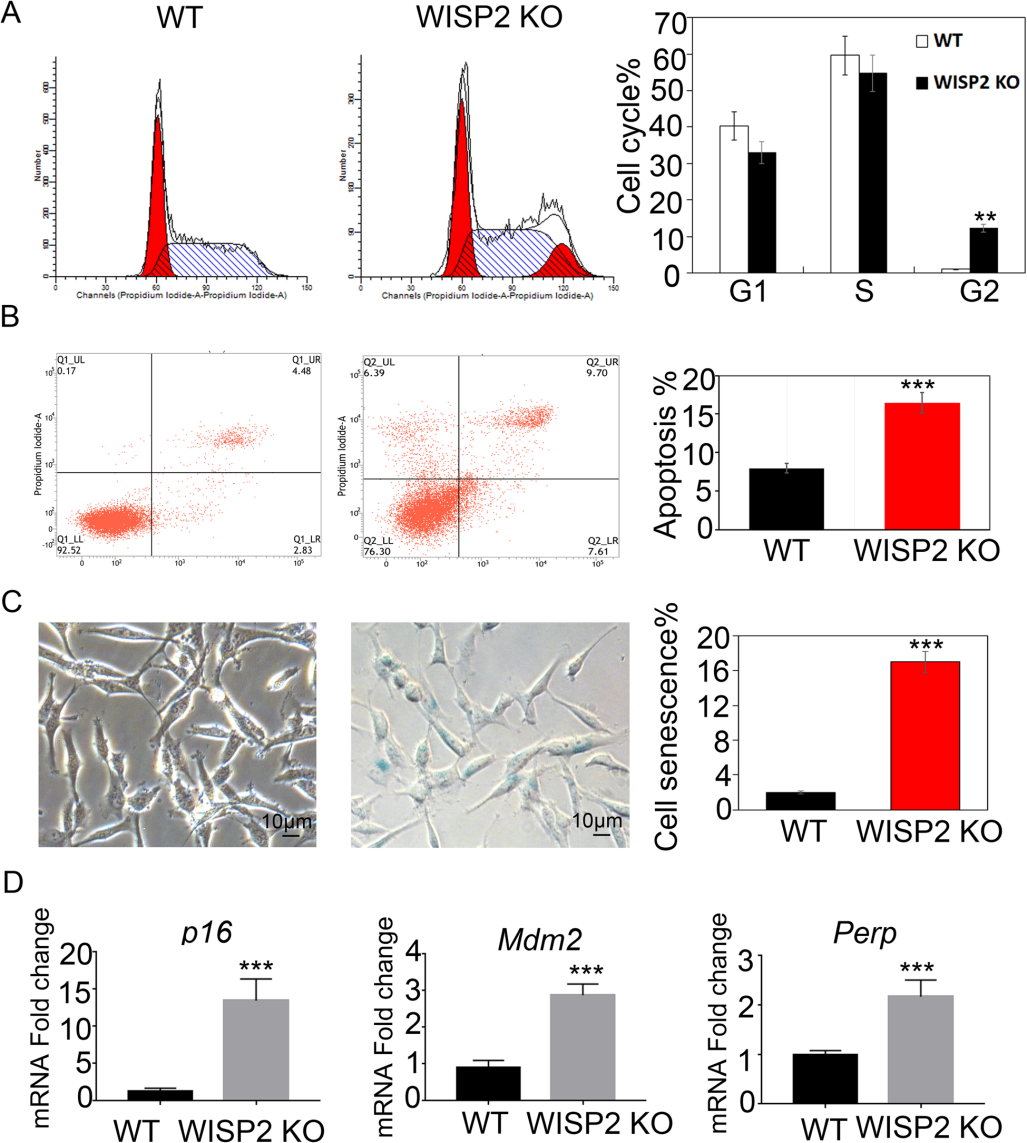
WISP2 deletion affects cell cycle and promotes apoptosis. **a** WISP2 deletion affected the cell cycle. ES-2 WT and WISP2 deletion cells were cultured overnight. PI (propidium iodine) staining followed by FACS detected the cell cycle stage. **b** WISP2 deletion promoted apoptosis. ES-2 WT and WISP2 deletion cells were cultured overnight, and 0.5 × 10^5^ cells were stained with PE Annexin V and analyzed via FACS within 1 h. **c** Loss of WISP2 promoted senescence. ES-2 WT and WISP2 deletion cells were cultured and stained for the senescence marker SA-β-gal. Quantification is shown in the right panel. **d** WISP2 deletion increased senescence-related gene expression. Expression of *p16*, *Perp,* and *Mdm2* mRNAs was determined via q-PCR. Data are expressed as the mean ± s.d. from three independent experiments. ** = *p* < 0.01; one-way ANOVA

FACS analysis revealed that WISP2 KO cells were arrested in the G2 phase of the cell cycle (Fig. 3a). Furthermore, we examined apoptosis of WISP2 deleted cells using a PE Annexin V apoptosis detection kit. Apoptosis was increased in WISP2 KO cells (Fig. 3b). In addition, staining with the senescence marker senescence-associated β-galactosidase (SA-β-gal) confirmed that WISP2 deleted cells displayed elevated SA-β-gal staining (Fig. 3c). These data indicate that WISP2 deletion results in cellular senescence. Furthermore, we analyzed the p16/pRB axes and p53, which are major senescence-triggering pathways [11]. We found that the levels of *p16*, *Mdm2* and *Perp* were increased in WISP2 deleted cells, verifying that knockout of WISP2 induces cellular senescence (Fig. 3d).

### WISP2 deletion inhibits cell proliferation by affecting the ERK signaling pathway

**Fig. 4:**
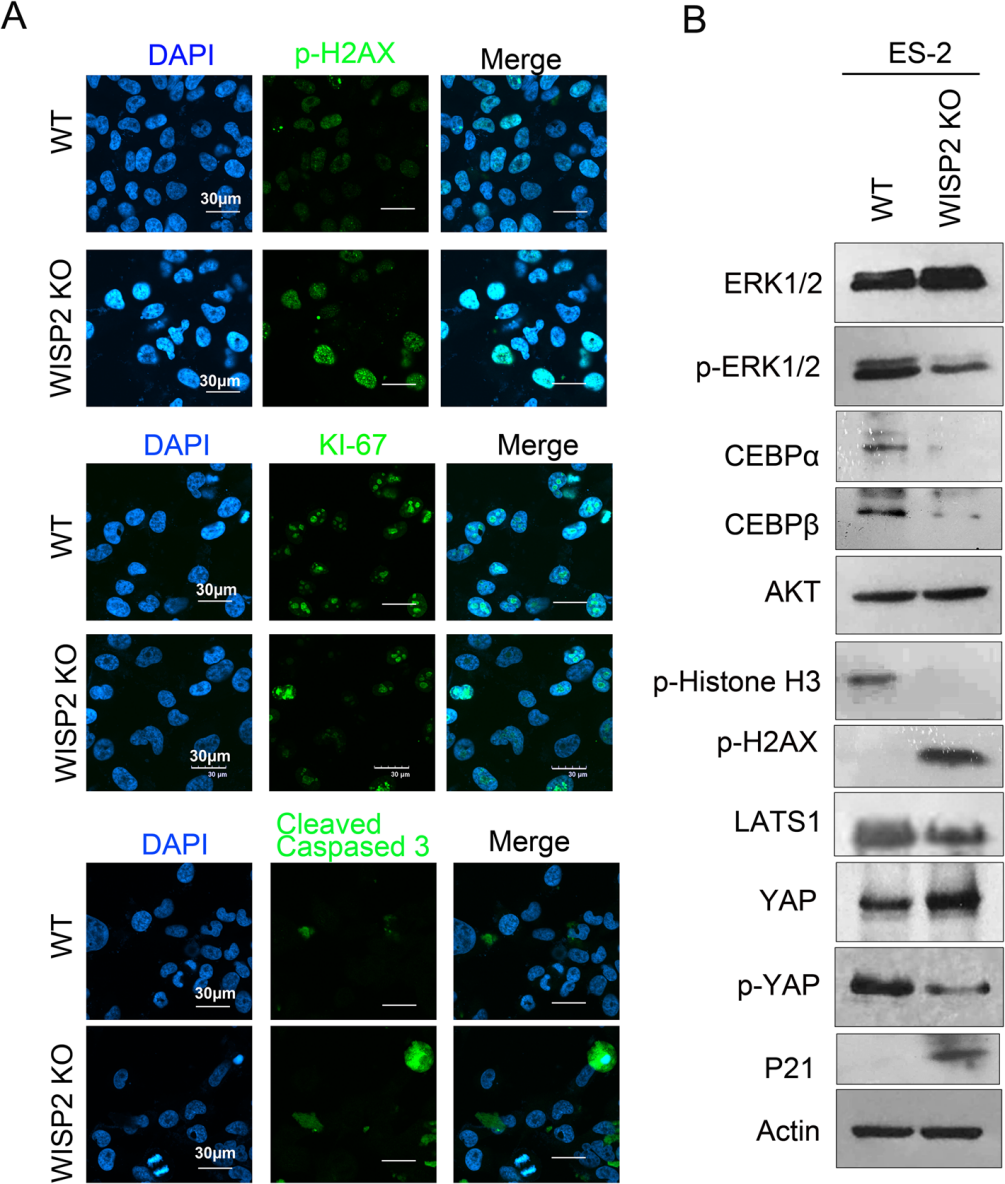
WISP2 deletion affects the ERK1/2 and Hippo signaling pathways. **a** WISP2 deletion inhibited the expression of the proliferation marker KI-67 and increased that of the apoptosis marker cleaved caspase-3. **b** ES-2 WT and WISP2 deletion cells were cultured for 24 h. Western blot was performed with the indicated antibodies

**Fig. 5:**
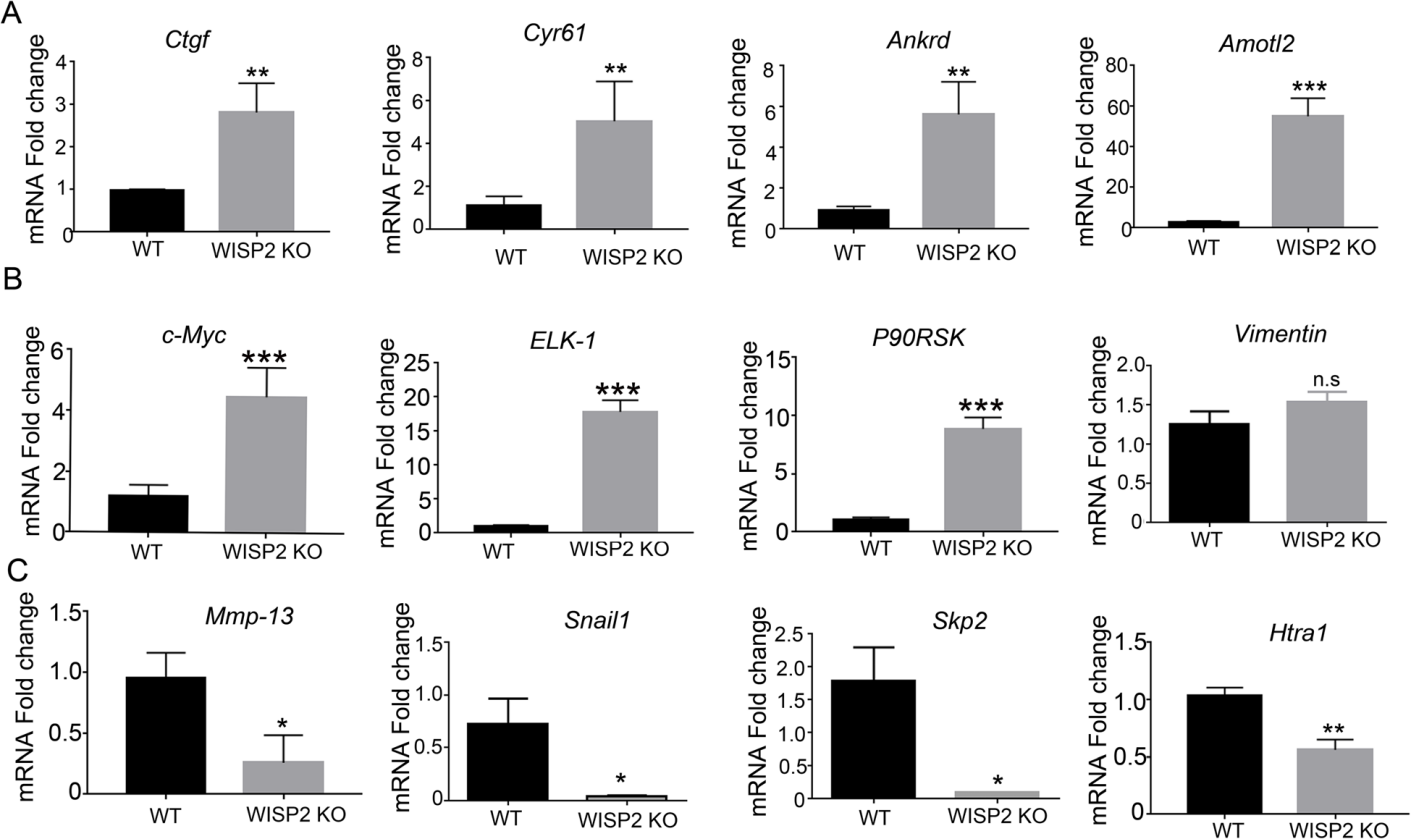
WISP2 deletion increases the expression of ERK1/2 and YAP target genes. **a** WISP2 deletion increased the expression of YAP/TAZ target genes *Ctgf, Cyr61, Ankrd,* and *Amotl2.***b** WISP2 deletion increased the expression of ERK1/2 target genes *c-Myc*, *Elk-1*, *P90RSK.***c** WISP2 deletion increased the expression of extracellular matrix-associated proteins. Results were obtained from experiments performed in triplicate. *p < 0.05, **p < 0.01, *** *p* < 0.001, ns = *p* > 0.05; Student’s t-test

To examine the mechanisms underlying the role of WISP2 in ovarian cancer cell growth regulation, we examined the cell proliferation marker KI-67 and the cell apoptosis marker cleaved caspase-3 via immunofluorescence assays. The results showed that WISP2 deletion significantly inhibited KI-67 expression in the nucleus and promoted apoptosis (Fig. 4a). Western blot analysis confirmed that the reduction in WISP2 protein expression inhibited p-ERK1/2, CEBPα, and CEBPβ. Furthermore, we observed that the deletion of WISP2 inhibited YAP phosphorylation (Fig. 4b) and increased the expression of the DNA damage marker p-H2AX. q-PCR analysis showed that the expression of the YAP target genes *Ctgf*, *Cyr61*, *Ankrd*, and *Amotl2* were increased in WISP2 KO cells (Fig. 5a). In addition, the expression of the ERK1/2 target genes *c-Myc*, *ELK-1*, *and P90RSK* also increased in WISP2 deletion cells, while that of the extracellular matrix-associated genes *Mmp13* and *snail* significantly decreased. Considering all of these findings, we propose that WISP2 deletion in ovarian cancer cells represses cell proliferation and increases senescence as well as apoptosis by affecting the ERK and Hippo signaling pathways.

### WISP2 deficiency inhibits tumor growth in vivo

**Fig. 6:**
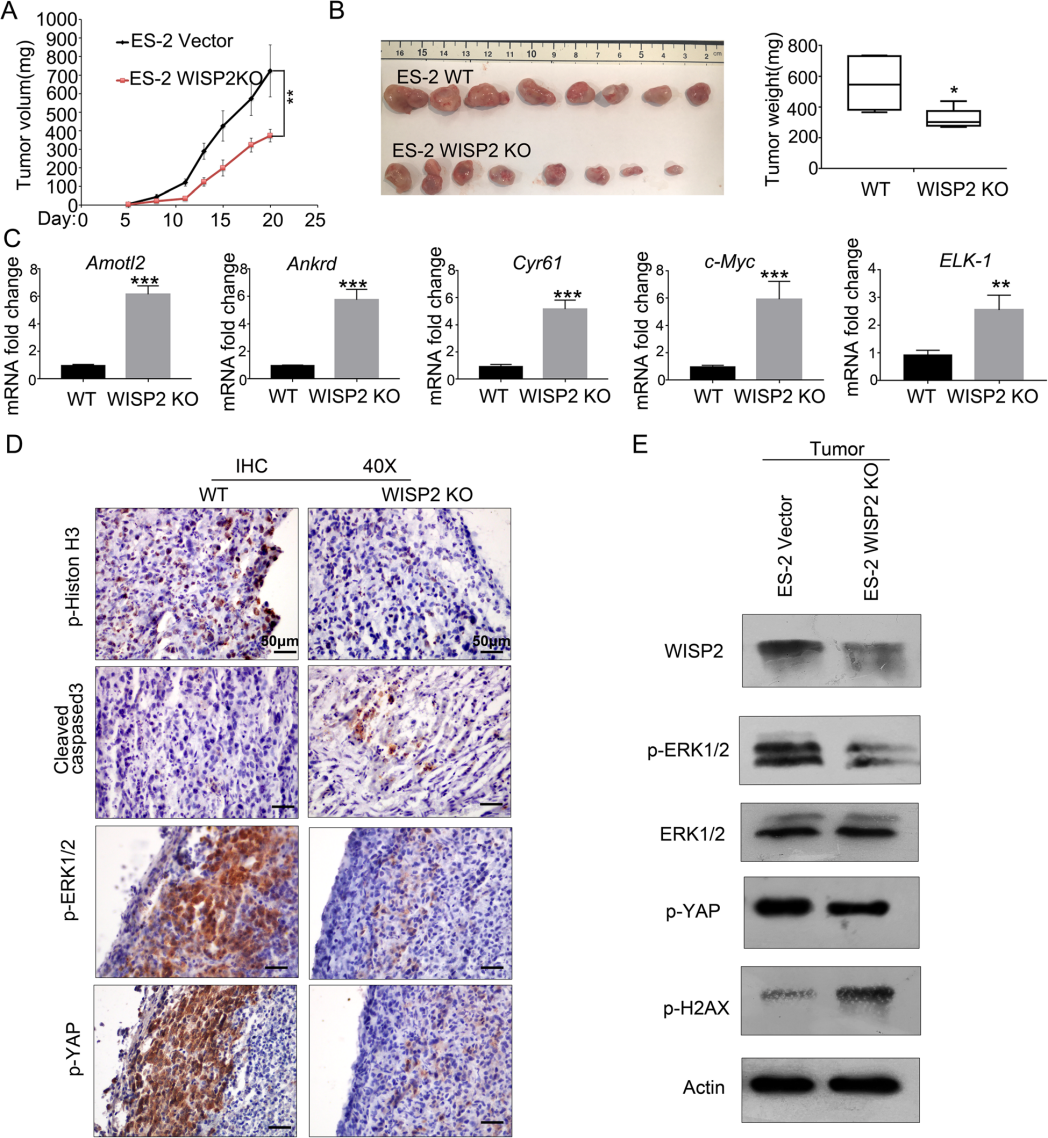
Loss of WISP2 inhibits ES-2 tumor growth in xenografted mice. **a-b** Deletion of WISP2 in ES-2 cells inhibited tumor growth in vivo. ES-2 WT and WISP2 deletion cells (1 × 10^6^ cells per each injection) were implanted subcutaneously into nude mice. Tumor volume was monitored, and tumor weight determined at the end of the experiments (*n* = 8). **c** Increased expression of YAP/TAZ and ERK1/2 target genes in WISP2 deleted tumors. RNA was isolated from tumor samples (panel B) and analyzed for target genes via RT-PCR. Data normalized relative to the corresponding value of WT tumors are expressed as the mean ± s.d (*n* = 3 tumors per group). *p < 0.05; **p < 0.01; *** *p* < 0.001; Student’s t-test. **d** Loss of WISP2 decreased cell proliferation and increased apoptosis in ES-2 tumors. Immunohistochemical staining for p-ERK1/2, p-YAP, cleaved caspase-3, and p-Histone H3 in representative tumor tissue; Scale bar, 50 μm. **e** Western blot detected protein expression in WISP2 deleted tumors. Tumor samples (panel B) were harvested and subjected to immunoblot analysis with the indicated antibodies

To evaluate the role of WISP2 in ES-2 tumor growth, we performed xenograft experiments in nude mice. Tumors grew slower in mice transplanted with WISP2 deficient ES-2 cells than in control mice (ES-2 WT group) (Figs. 6a and b). q-PCR results showed that the YAP (*Cyr61*, *Amotl2*, and *Ankrd1*) and ERK1/2 target genes (*c-Myc* and *ELK-1*) were elevated in WISP2 deficient tumor samples (Fig. 6c). In addition, we found that the level of cleaved caspase-3 was increased, whereas that of p-Histone H3, p-ERK1/2, and p-YAP were decreased in WISP2 deficient tumors (Fig. 6d). Western blotting confirmed the reduction in WISP2 protein, YAP S127 phosphorylation, and ERK1/2 phosphorylation in tumors derived from WISP2 KO cells (Fig. 6e). Collectively, our results indicate that WISP2 deletion inhibits ovarian cancer cell growth in vitro and in vivo*.*

## Discussion

WISP2 reportedly governs the expression of several genes in human cancer cells, such as those of breast, colon, gastric, pancreatic, and esophageal cancers [12–15]. Clinical studies have shown that the expression profile and role of WISP2 in various cancers may differ, and such inconsistencies, which have been observed between multiple cancers, have raised uncertainty concerning the role of WISP2 in carcinogenesis. For example, in MCF-7 cells, a proliferative role of WISP2 has been reported in which silencing WISP2 inhibited cell proliferation induced by serum and epidermal growth factor, whereas it acts as a growth repressor in prostate cancer cells [16, 17]. In addition, WISP2 likely plays a preventive role in the progression of pancreatic cancer by participating in morphological alterations from mesenchymal to epithelial transition (MET) of pancreatic adenocarcinoma [12] and breast cancer cells [18]. However, the role of WISP2 in ovarian cancer cells remains unelucidated.

The present study indicates that although WISP2 protein levels were elevated in different ovarian cancer tissues and cell lines, it was minimally detectable in normal ovarian epithelial cells. This suggests that elevated WISP2 levels may play a specific role in human ovarian cancer progression. Deletion of WISP2 inhibited ovarian cancer cell proliferation and clone number and induced cell apoptosis as well as senescence. Molecular mechanism studies have indicated that WISP2 deletion inhibited ERK1/2 and YAP phosphorylation in vivo and in vitro and subsequently increased p16 and p21 accumulation.

The mitogen-activated protein kinase/ERK (MAPK/ERK) and Hippo pathways are reportedly associated with cell proliferation, differentiation, migration, senescence, and apoptosis [19, 20]. Previous studies have shown that alteration in the MAPK/ERK pathway is strongly implicated in ovarian cancer pathogenesis [21, 22]. Dang et al. [23] reported that metformin combined with cisplatin inhibited cell viability and promoted apoptosis of human ovarian cancer cells by inactivating ERK1/2. Xia et al. [24] reported that YAP, which promotes ovarian cancer cell tumorigenesis, was indicative of a poor prognosis for ovarian cancer patients. A previous study of ours demonstrated that the death-associated protein 6 (DAXX) could induce ovarian cancer ascites formation by activating the ERK signaling pathway [25]. Large tumor suppressor (LATS)1/2 deletion inhibited colon cancer cells by promoting YAP transcription [10]. In the current study, we found that WISP2 deletion inhibited the expression of p-ERK and p-YAP, which induced the expression of ERK1/2 and YAP target genes, such as *Ankrd, Amotl2, c-Myc, ELK-1,* and *p90RSK*. Thus, we concluded that the effect of WISP2 inhibition on ES-2 and H08910 cells may, at least partly, be due to the downregulation of p-ERK1/2 and p-YAP.

In addition, WISP2 was reported as an extracellular matrix (ECM)-associated adhesion protein as it contains binding sites for both ECM and cell surface proteins. WISP2 regulated cell growth, migration, and metastasis by regulating EMT and inhibiting matrix metalloproteinase (MMP)-9 and MMP-2 via ERK in gastric cancer cells [26]. In the present study, WISP2 deletion decreased the expression of p-ERK1/2. Thus, WISP2 may inhibit the proliferation and induce apoptosis by suppressing ERK1/2 signaling in ovarian cancer cells. E-cadherin is a key molecule involved in EMT and cell invasion [27, 28]. WISP2 regulated cell migration and invasion by affecting Snail and E-cadherin in breast cancer cells [29]. Our study also demonstrated that WISP2 deletion decreased Snail and MMP-13 levels in ovarian cancer cells. Therefore, WISP2 may promote invasion and migration by regulating EMT in ovarian cancer cells.

Our data on WISP2 expression in ovarian cancer cells have clinical and therapeutic implications. WISP2 deletion suppressed the tumorigenic capacity of ovarian cancer cells in vivo and in vitro. Thus, small molecules capable of inhibiting WISP2 activity or accumulation may have therapeutic potential in ovarian cancer patients. These results indicate that WISP2 expression may play a key role in initiating ovarian cancer and, therefore, be used as an early diagnostic marker for ovarian cancer.

## Conclusions

WISP2 deletion inhibits ovarian cancer cell proliferation by affecting ERK and YAP signaling pathways.

## Methods

### Cell culture and CRISPR

HO8910 and ES-2 ovarian cancer cells were cultured in DMEM/RPMI1640 supplemented with 10% FBS and 1% penicillin-streptomycin solution at 37 °C under a humidified 5% CO_2_ atmosphere.

WISP2 deletion cells were generated via CRISPR genomic editing technology. These plasmids were then transfected into HO8910 and ES-2 cells. Twenty-four hours following transfection, transfected cells were enriched via puromycin selection for 3 d and sorted on 96-well plates with only a single cell per well. The clones were screened via western blot using WISP2 antibody (1:2000, Abcam).

*Wisp2* sgRNA sequence was as follows:

*Wisp2–1*: GCTGTGAGGTGAATGGCCGC.

*Wisp2–2*: TTGCCGGCTGCATCACTGCC.

### Soft agar colony formation assay

One-milliliter layers of 0.5% agar were prepared in a 35 mm cell culture dish. Cells were suspended in 1 ml of 0.35% agar containing 1 × cell culture medium and 10% FBS and poured over these layers. The final cell concentration in each culture was 0.5 × 10^3^ cells/ml. Triplicate cultures were used for each experiment. Plates were placed in a 5% CO_2_ humidified incubator at 37 °C. Colonies were counted 2–3 weeks after plating using an Omnicon FAS II Image Analysis System.

### Wound-healing assay

ES-2 WT and WISP2 deleted cells were grown in DMEM supplemented with 10% FBS until confluence was reached. The medium was then changed to fresh serum-free medium, and the cell monolayers were scraped in a straight line using a P-10 pipette tip to create a scratch. The plates were photographed at 0 and 24 h using a phase-contrast inverted microscope (Nikon Ti, Nikon Corp.).

### Transwell migration assay

Twenty-four-well tissue culture plate inserts with 8 μm-pore filters and BioCoat Matrigel (BD Biosciences, Bedford, MA, USA) were used to assess the migration and invasive potential of ES-2 and HO8910 WT and WISP2 deleted cells. The cells were suspended in serum-free medium and then added to a transwell (100 μl cell suspension/well at a concentration of 0.5–1 × 10^5^ cells/ml). After incubation for 24 h at 37 °C, cells at the upper surface of the transwell were removed using cotton swabs. Migrated cells that had attached to the lower surface were stained using hematoxylin and eosin. Transwells were rinsed with water and air-dried. Positive cells were quantified using Image-Pro Plus 6.0 software.

### PE Annexin V apoptosis detection

ES-2 WT and WISP2 deficient cells were cultured in 6-well plates overnight. Cells were washed twice with cold PBS and resuspended in 1 × Binding Buffer at a concentration of 1 × 10^6^ cells/ml. Next, 1 × 10^5^ cells were transferred to a 5 ml culture tube, treated with 5 μl of PE Annexin V and 7-amino-actinomycin D, gently vortexed, and incubated for 15 min at 25 °C in the dark. After incubation, 400 μl of 1 × Binding Buffer was added to each tube, and cells were analyzed via flow cytometry within 1 h.

### Mice and xenograft models

Mice (*n* = 8) were housed under standard conditions with a 14 h/10 h light/dark cycle and provided with food and water ad libitum*.* All animal protocols were in accordance with the NIH Guide for the Care and Use of Laboratory Animals. To assess cancer cell proliferation in vivo, we subcutaneously transplanted ES-2 WT or WISP2 deficient cells (1 × 10^6^) into both back flanks of 8-week-old female nude mice. Three weeks later, primary tumor masses were collected from athymic nude mice, fixed in 4% paraformaldehyde, and embedded in paraffin.

### Immunohistochemical (IHC) analysis

Primary tumor masses were excised and fixed in 4% paraformaldehyde in PBS overnight. For immunochemistry related studies, sections were deparaffinized, rehydrated with xylene and a descending alcohol gradient, and incubated in 0.3% H_2_O_2_. Following antigen retrieval using 10 mM sodium citrate (pH 6.0), sections were incubated with anti-WISP2, anti-p-ERK1/2, anti-p-YAP, anti-p-Histone H3, anti-cleaved caspase-3 antibodies (Cell Signaling Technology, 1:200) using a Vector ABC kit (Vector Laboratories) at room temperature for 1 h. Afterward, the sections were allowed to react with biotin-labeled secondary antibodies for 30 min. Staining was performed using the Vectastain ABC kit and 3,3′-diaminobenzidine (DAB) peroxidase substrate kit (Vector Laboratories, Burlingame, CA, USA).

### Immunofluorescence analysis

Cells were cultured in a 24-well plate overnight, washed with PBS, and fixed for 10 min at room temperature with 4% paraformaldehyde in PBS. Cells were permeabilized with 0.3% Triton X-100 in PBS, incubated with the blocking buffer (PBST containing 5% bovine serum albumin), and sequentially probed with anti-p-H2AX, anti-Ki-67, and anti-cleaved caspases-3 antibodies (Cell Signaling Technology, 1:200) and 488-conjugated secondary antibodies (Molecular Probes). Slides were mounted using a VectaShield with 4′, 6-diamidino-2-phenylindole (DAPI, Vector Laboratories). Digital images were acquired using a laser scanning confocal microscope with 6–100 × magnification.

### Western blot analysis

Total proteins were isolated from the cell extracts, and 30 μg of protein were separated by SDS-PAGE and transferred to polyvinylidene difluoride (PVDF) membranes (Millipore, Bedford, MA, USA). After probing with primary antibodies, membranes were washed in Tris-buffered saline containing 0.05% Tween-20 (TBST) and incubated with horse-radish peroxidase-linked secondary antibodies. Finally, the obtained bands were detected using an Enhanced Chemiluminescence Detection Kit (Millipore, Bedford, MA, USA).

The primary antibodies used were as follows:

**Table:** 

Antibodies	Source	Indentifier
WISP2	Abcam	Cat#:31317
LATS1	Cell Signaling	Cat#:3477
YAP	Cell Signaling	Cat#:14074
YAPS127	Cell Signaling	Cat#:4911
LATS2	Cell Signaling	Cat#:5888
ERK1/2	Cell Signaling	Cat#:4695
p-ERK1/2	Cell Signaling	Cat#:4370
Actin	Abcam	Cat#:ab3280
AKT	Cell Signaling	Cat#:9272
p-AKT	Cell Signaling	Cat#:4058
PARP	Cell Signaling	Cat#:9532
cleaved caspase-3	Cell Signaling	Cat#:9664
KI-67	Cell Signaling	Cat#:9129
p-Histone H3	Cell Signaling	Cat#:9701

### RNA extraction and real-time RT-PCR analysis

Total RNA was extracted using TRIZOL, according to the manufacturer’s instructions. Real-time PCR analysis was performed using a KAPA SYBR FAST qPCR kit (Kapa Biosystems, USA) and an Applied 7300 Real-Time PCR System. Relative mRNA levels were determined by normalizing the obtained expression levels to endogenous GAPDH mRNA levels using Microsoft EXCEL. The relative transcript levels of the control sample were set at 1 and compared with the transcript levels of the other samples. Quantitative RT-PCR reactions were performed in triplicate. The following primers were used to amplify target genes:

*Gapdh*: 5′-GCCTGGAGAAACCTGCCAAGTATG-3′ and 5′-GAGTGGGAGTTGCTGTTGAAGTCG-3′;

*Ctgf*: 5′-AGCTGACCTGGAGGAAAACA-3′ and 5′-GACAGGCTTGGCGATTTTAG-3′;

*Cyr61*: 5′-GCTCAGTCAGAAGGCAGACC-3′ and 5′-GTTCTTGGGGACACAGAGGA-3′;

*Amotl2*: 5′-AGGAGAAGAGTTGCCCACCTATGAG-3′ and 5′-TCGAAGAGCTTCATCCTGTCGC-3′;

*p90RSK*: 5′-CAGAGACCTCAAGCCTGAGAAC-3′ and 5′-CCACCAGTCCGCACTATGGG-3′;

*c-Myc*: 5′-ACCAGAGTTTCATCTGCGACCC-3′ and5’-TGGAGGTGGAGCAGACGCTG-3′.

*Elk1*: 5′-AGGCAATGGCCACATCATCTC-3′ and 5′-CGCTCCCTTGCGGATGATG-3′;

*Mdm2*: 5′-CCTGGCTCTGTGTGTAATAAG-3′ and 5′-ATCCAACCAATCACCTGAATG-3′;

*Perp*: 5′-GGCTTCATCATCCTGGTGAT-3′ and 5′-ACAGCAGCCAAGGCAAGGAG-3′;

*Snai1*: 5′-AGAGTTTACCTTCCAGCAGCC-3′ and 5′-GGACAGAGTCCCAGATGAGC-3′;

*Vimentin-1*: 5′-TACATCGACAAGGTGCGCTT-3′ and 5′-TCGTTGGTTAGCTGGTCCAC-3′;

*Skp2*: 5′-TTGCGCATGTGTCAGAGACC-3′ and 5′-AGGTGTTGGAGGTAGTTGAGC-3′;

*p16*: 5′- CTGCCCAACGCACCGAATAG-3′ and 5′- ACCACCAGCGTGTCCAGGAA-3′.

*Mmp13*: 5′- AAGGAGCATGGCGACTTC-3′ and 5′- TGGCCCAGGAGGAAAAGC-3′.

*Htra1*: 5′-GGGACTGGTCGTGTTTGTGC-3′ and 5′-CATTGACCTTTGGGTGCTGACT-3′.

### Statistical analyses

All in vitro assays were performed in triplicate. Groups were compared using two-tailed t-tests or ANOVA via the statistical program GraphPad Prism (GraphPad Prism, San Diego, CA, USA). Statistical significance was set at *p* < 0.05.

## Data Availability

All data generated or analyzed during this study are included in the present article. The datasets supporting the conclusions of this article are included in the article.

## Abbreviations

CEBP: CCAAT/enhancer-binding protein
CRISPR/Cas9: Clustered regularly-interspaced short palindromic repeats/Cas9
CTGF: Connective tissue growth factor
Cyr61: Cysteine-rich 61
DMEM: Dulbecco’s Modified Eagle’s Medium
DNMT1: DNA methyltransferase 1
ERK: Extracellular signal-related kinase
FACS: Fluorescent-activated cell sorting
LATS: Large tumor suppressor
MAPK: Mitogen-activated protein kinase
MMP: Matrix metalloproteinase
mOSE: Mouse ovarian surface epithelium
Skp2: S-phase kinase-associated protein 2
WISP2: Wnt-inducible signaling protein 2
YAP: Yes-associated protein

## References

[1] Siegel RL, Miller KD, Jemal A (2019). Cancer statistics, 2019. CA Cancer J Clin.

[2] Stewart C, Ralyea C, Lockwood S (2019). Ovarian cancer: an integrated review. Semin Oncol Nurs.

[3] Brigstock DR, Goldschmeding R, Katsube KI, Lam SC, Lau LF, Lyons K (2003). Proposal for a unified CCN nomenclature. Mol Pathol.

[4] Leask A, Abraham DJ (2006). All in the CCN family: essential matricellular signaling modulators emerge from the bunker. J Cell Sci.

[5] Perbal B (2013). CCN proteins: a centralized communication network. J Cell Commun Signal.

[6] Li J, Ye L, Owen S, Weeks HP, Zhang Z, Jiang WG (2015). Emerging role of CCN family proteins in tumorigenesis and cancer metastasis. Int J Mol Med.

[7] Inadera H, Shimomura A, Tachibana S (2009). Effect of Wnt-1 inducible signaling pathway protein-2 (WISP-2/CCN5), a downstream protein of Wnt signaling, on adipocyte differentiation. Biochem Biophys Res Commun.

[8] Russo JW, Castellot JJ (2010). CCN5: biology and pathophysiology. J Cell Commun Signal.

[9] Berger T, Sidhu P, Tang S, Kucera H (2019). Are testicular cortisol and WISP2 involved in estrogen-regulated Sertoli cell proliferation?. Anim Reprod Sci.

[10] Pan WW, Moroishi T, Koo JH, Guan KL (2019). Cell type-dependent function of LATS1/2 in cancer cell growth. Oncogene.

[11] Sharpless NE, Sherr CJ (2015). Forging a signature of in vivo senescence. Nat Rev Cancer.

[12] Frewer KA, Sanders AJ, Owen S, Frewer NC, Hargest R, Jiang WG (2013). A role for WISP2 in colorectal cancer cell invasion and motility. Cancer Genomics Proteomics.

[13] Dhar G, Mehta S, Banerjee S, Gardner A, McCarty BM, Mathur SC (2007). Loss of WISP-2/CCN5 signaling in human pancreatic cancer: a potential mechanism for epithelial-mesenchymal-transition. Cancer Lett.

[14] Ji J, Jia S, Jia Y, Ji K, Hargest R, Jiang WG (2015). WISP-2 in human gastric cancer and its potential metastatic suppressor role in gastric cancer cells mediated by JNK and PLC-γ pathways. Br J Cancer.

[15] Chai DM, Qin YZ, Wu SW, Ma L, Tan YY, Yong X (2019). WISP2 exhibits its potential antitumor activity via targeting ERK and E-cadherin pathways in esophageal cancer cells. J Exp Clin Cancer Res.

[16] Inadera H, Hashimoto S, Dong HY, Suzuki T, Nagai S, Yamashita T (2000). WISP-2 as a novel estrogen-responsive gene in human breast cancer cells. Biochem Biophys Res Commun.

[17] Ray G, Banerjee S, Saxena NK, Campbell DR, Van Veldhuizen P, Banerjee SK (2005). Stimulation of MCF-7 tumor progression in athymic nude mice by 17beta-estradiol induces WISP-2/CCN5 expression in xenografts: a novel signaling molecule in hormonal carcinogenesis. Oncol Rep.

[18] Haque I, Banerjee S, De A, Maity G, Sarkar S, Majumdar M (2015). CCN5/WISP-2 promotes growth arrest of triple-negative breast cancer cells through accumulation and trafficking of p27(kip1) via Skp2 and FOXO3a regulation. Oncogene..

[19] Yu P, Ye L, Wang H, Du G, Zhang J, Zhang J (2015). NSK-01105 inhibits proliferation and induces apoptosis of prostate cancer cells by blocking the Raf/MEK/ERK and PI3K/Akt/mTOR signal pathways. Tumour Biol.

[20] Yu FX, Zhao B, Guan KL (2015). Hippo pathway in organ size control, tissue homeostasis, and cancer. Cell..

[21] Liu S, Zha J, Lei M (2018). Inhibiting ERK/Mnk/eIF4E broadly sensitizes ovarian cancer response to chemotherapy. Clin Transl Oncol.

[22] Hu Y, Yang L, Yang Y, Han Y, Wang Y, Liu W (2016). Oncogenic role of mortalin contributes to ovarian tumorigenesis by activating the MAPK-ERK pathway. J Cell Mol Med.

[23] Dang JH, Jin ZJ, Liu XJ, Hu D, Wang J, Luo Y (2017). Metformin in combination with cisplatin inhibits cell viability and induces apoptosis of human ovarian cancer cells by inactivating ERK 1/2. Oncol Lett.

[24] Xia Y, Chang T, Wang Y, Liu Y, Li W, Li M (2014). YAP promotes ovarian cancer cell tumorigenesis and is indicative of a poor prognosis for ovarian cancer patients. PLoS One.

[25] Liu SB, Lin XP, Xu Y, Shen ZF, Pan WW (2018). DAXX promotes ovarian cancer ascites cell proliferation and migration by activating the ERK signaling pathway. J Ovarian Res.

[26] Barreto SC, Ray A, Ag EP (2016). Biological characteristics of CCN proteins in tumor development. J BUON.

[27] Prieto-García E, Díaz-García CV, García-Ruiz I, Agulló-Ortuño MT (2017). Epithelial-to-mesenchymal transition in tumor progression. Med Oncol.

[28] Wong SHM, Fang CM, Chuah LH, Leong CO, Ngai SC (2018). E-cadherin: its dysregulation in carcinogenesis and clinical implications. Crit Rev Oncol Hematol.

[29] Banerjee SK, Banerjee S (2012). CCN5/WISP-2: a micromanager of breast cancer progression. J Cell Commun Signal.

